# Stimulus-level interference disrupts repetition benefit during task switching in middle childhood

**DOI:** 10.3389/fnhum.2013.00841

**Published:** 2013-12-05

**Authors:** Frini Karayanidis, Sharna Jamadar, Dearne Sanday

**Affiliations:** ^1^Functional Neuroimaging Lab, School of Psychology, University of NewcastleCallaghan, NSW, Australia; ^2^Centre for Translational Neuroscience and Mental Health, University of Newcastle and Hunter Medical Research InstituteCallaghan, NSW, Australia; ^3^Monash Biomedical Imaging and School of Psychology and Psychiatry, Monash UniversityMelbourne, VIC, Australia

**Keywords:** cognitive control, development, task switching, event-related potentials

## Abstract

The task-switching paradigm provides a powerful tool to measure the development of core cognitive control processes. In this study, we use the alternating runs task-switching paradigm to assess preparatory control processes involved in flexibly preparing for a predictable change in task and stimulus-driven control processes involved in controlling stimulus-level interference. We present three experiments that examine behavioral and event-related potential (ERP) measures of task-switching performance in middle childhood and young adulthood under low and high stimulus interference conditions. Experiment 1 confirms that our new child-friendly tasks produce similar behavioral and electrophysiological findings in young adults as those previously reported. Experiment 2 examines task switching with univalent stimuli across a range of preparation intervals in middle childhood. Experiment 3 compares task switching with bivalent stimuli across the same preparation intervals in children and young adults. Children produced a larger RT switch cost than adults with univalent stimuli and a short preparation interval. Both children and adults showed significant reduction in switch cost with increasing preparation interval, but in children this was caused by greater increase in RT for repeat than switch trials. Response-locked ERPs showed intact preparation for univalent, but less efficient preparation for bivalent stimulus conditions. Stimulus-locked ERPs confirmed that children showed greater stimulus-level interference for repeat trials, especially with bivalent stimuli. We conclude that children show greater stimulus-level interference especially for repeat trials under high interference conditions, suggesting weaker mental representation of the current task set.

## Introduction

Cognitive control involves a range of psychological processes that regulate conscious thought and behavior, including working memory, inhibition, and cognitive flexibility (Miyake et al., 2000; Diamond, 2013). These higher order cognitive functions and the frontal brain areas that support them show protracted development, reaching full maturation as late as the third decade of life (e.g., Lebel et al., 2008). Poor cognitive control is associated with atypical development as reflected in a range of neurodevelopmental disorders such as attention-deficit hyperactivity disorder, oppositional defiance disorder, and conduct disorder (Makris et al., 2009; Seguin, 2009) and is a risk factor for poor social integration and low resilience (Greenberg, 2006). Therefore, an understanding of the processes that mediate typical development of cognitive control is essential to determining how these mechanisms are disrupted in atypical development.

The task-switching paradigm provides a powerful tool for measuring two aspects of cognitive control: *advance preparation*, i.e., the ability to efficiently anticipate, select, and prepare a task set; and *task-set implementation*, i.e., the ability to implement action plans and resist interference (see Karayanidis et al., 2010 for review). Participants switch between two simple tasks using a fixed task sequence (alternating runs, Rogers and Monsell, 1995) or a random sequence of task cues (cued-trials, Meiran et al., 2000). Reaction time (RT) is longer on switch relative to repeat trials and this “*switch cost*” reduces as the time to prepare in advance of a switch trial increases (e.g., Rogers and Monsell, 1995; Meiran, 2000; Karayanidis et al., 2003). However, even with very long preparation intervals (i.e., response-stimulus interval (RSI) in alternating runs paradigms and cue-stimulus interval (CSI) in cued trials paradigms), a *residual switch cost* remains, suggesting that advance preparation is not sufficient to fully equate switch and repeat trials (Allport et al., 1994; Rogers and Monsell, 1995). This residual switch cost has been attributed to incomplete advance preparation before the onset of the stimulus under some task conditions (Verbruggen et al., 2007), sustained interference from the previously active task set (e.g., Mayr and Keele, 2000), difficulty re-activating a currently irrelevant task set (e.g., Allport et al., 1994; Mayr and Keele, 2000) and/or intermittent failure to engage the correct task set (De Jong, 2000).

### Behavioral studies of task-switching in development

A number of behavioral studies have examined developmental changes in task-switching performance. *General switch cost*^1^ —that is, performance difference between single-task blocks (i.e., repeating the same task throughout the block) and mixed-task blocks (i.e., alternating between task switch and task repeat trials within the same block) has been found to decrease from middle childhood into young adulthood (Kray et al., 2004, 2008; Reimers and Maylor, 2005; Karbach and Kray, 2007). However, general switch cost may arise from a number of underlying processes that differ between single-task and mixed-task blocks, including task difficulty, arousal, attention, working memory, task-set selection, and task-set switching (Rogers and Monsell, 1995). It is therefore useful to differentiate between *mixing cost*, the effect of repeating the same task in a single-task block vs. repeat trials presented in a mixed-task block (“mixed-repeat” trials), and *switch cost*, the effect of repeating vs. switching task within a mixed-task block (often also referred to as *local* or *specific* switch cost).

Mixing cost has been attributed to additional processes required for selecting and maintaining task sets in working memory (Los, 1996) and/or resolving stimulus ambiguity (Mayr, 2001) in mixed-task blocks. Switch cost is believed to reflect the efficiency with which one is able to inhibit the irrelevant task rule and engage the relevant task rule on any given trial. Given the protracted development of cognitive control, we would expect switch cost to be a strong contributor to the developmental trends in general switch cost. Yet, despite consistent evidence for a gradual decline in *mixing* cost with increasing age (Kray et al., 2004, 2008; Crone et al., 2006; Karbach and Kray, 2007), developmental patterns for *switch*-cost are more complex.

Studies using long preparation intervals report no differences in switch cost between children and adults (Kray et al., 2004; Reimers and Maylor, 2005; see also Karbach and Kray, 2007), whereas studies using paradigms that offer little or no opportunity for advance preparation report larger switch cost in children. For instance, Crone et al. (2006) reported that RT switch cost, but not mixing cost, was larger for 7–8 year-olds than both 10–12 year-olds and adults. Davidson et al. (2006) reported higher error but not RT switch cost in 6–13 year-olds than in adults. Using alternating runs designs, both Kray et al. (2008) and Huizinga et al. (2006) reported large decline in RT switch cost from middle childhood to adulthood. Stoet and López (2013) showed that switch cost reduced from 9 to 16 years but the slope of the age effect was not affected by either task difficulty or stimulus congruity. Together, these results suggest that the ability to engage the relevant task rule and/or disengage the irrelevant task rule continues to mature into adolescence, especially when not given adequate time to prepare.

Cue-trials task-switching paradigms also suggest greater stimulus-level interference in children. Cepeda et al. (2001) showed that young children had a larger RT switch cost and greater reduction in RT switch cost with task practice than adults. Increasing CSI produced a significant reduction in RT switch cost for both children and adults, whereas increasing RCI reduced RT switch cost in adults only. These findings suggest that, given sufficient time, children can efficiently prepare for a predictable switch trial but that the representation of the previously active task rules decays more slowly in children than in adults, which can result in greater stimulus-level interference. Using a similar paradigm to Cepeda, but with both CSI and RCI set to zero, Gupta et al. (2009) reported a non-linear decline in RT switch cost over 6–11 years of age, with the rate of decline varying as a function of both response repetition and stimulus-response(S-R) compatibility. Cragg and Nation (2009) also found that stimulus-level interference has a greater impact on switch cost in children. Young (5–8 year-olds) children had larger RT switch cost than older (9–11 year-olds) children in a cued task-switching paradigm. Paradoxically, greater stimulus-level interference increased switch cost for older but not younger children. However, this resulted from younger children showing a disproportionate increase in RT for *repeat trials* in the high interference condition. So, young children benefitted less from task repetition under high stimulus interference.

Overall, behavioral studies show evidence for larger switch cost in childhood with the size of the effect and the age of achievement of adult performance level varying as a function of task parameters and measures. There is some evidence for greater stimulus-level interference in younger children. However, the processes that underlie this effect on task-switching performance remain to be defined.

### ERP studies of task-switching in development

Conventional behavioral measures, like mean response time (RT) and error rate represent the endpoint of decision making and do not offer direct insight about the temporal evolution of the processes leading up to the decision. This limitation is particularly pertinent with respect to processes contributing to advance preparation, as these processes are covert and are likely to involve multiple overlapping processes that can be difficult to disentangle using RT and error rate alone. The temporal resolution of event-related potentials (ERPs) makes them particularly suitable for examining advance preparation and task-set implementation in task-switching.

In adults, ERPs locked to the onset of the preparation interval typically show a larger posterior positivity for switch than repeat trials (see Karayanidis et al., 2010 for review). This differential *switch-positivity* emerges as early as 150 ms after preparation onset and is often fully resolved prior to stimulus onset in optimal conditions (e.g., long preparation interval, valid task-specific cue). The switch-positivity is believed to index anticipatory control processes related to preparing for an upcoming switch trial. It consists of a number of subcomponents that are sensitive to shifting attention between stimulus sets and S-R mappings and is affected by the degree to which the upcoming stimulus is prone to interference. Stimulus-locked ERPs show a larger central negativity and smaller posterior positivity for switch relative to repeat trials, sometimes emerging as early as 150 ms post-stimulus (e.g., Karayanidis et al., 2003; Kieffaber and Hetrick, 2005; Nicholson et al., 2005). These effects are linked to target-driven control processes involved in overcoming interference due to task-set inertia or S-R priming as conceptualized by Allport and colleagues (e.g., Jamadar et al., 2010b; Wylie and Allport, 2000).

To date, only one study has examined ERP correlates of task-switching in children. Manzi et al. (2011) examined cue-locked ERPs in children (9–11 years), adolescents (13–15 years) and young adults (20–27 years) using an explicitly cued task-switching paradigm with single-task and mixed-task blocks. Children but not adolescents produced a larger RT mixing cost than adults but the age effect was eliminated when CSI increased from 600 to 1200 ms. In contrast, RT switch cost did not differ with age. In cue-locked ERPs, the *“mixing-positivity”* or the increase in positivity for repeat trials in mixed-task vs. single-task blocks emerged later and was larger in children, suggesting that children engage in more effortful preparation in mixed-task than in single-task blocks relative to adults. At the shorter CSI of 600 ms, the switch-positivity emerged later and had a shorter duration in children than adults (adults: 300–900 ms, children: 500–700 ms). At the long CSI (1200 ms), children showed no differential preparation for switch and repeat trials. This pattern of results suggests that children engage in more effortful preparation for mixed-task blocks but may prepare equally for switch and repeat trials. Stimulus-locked ERPs were not examined.

### Present study

In the present study, we systematically examine behavioral and electrophysiological measures of task-switching performance in middle childhood (6–12 year-olds) compared to young adulthood across a range of RSI using variations of the alternating runs paradigm (Rogers and Monsell, 1995; Karayanidis et al., 2003). We examine both response-locked and stimulus-locked ERPs to dissociate the contribution of preparatory and target-driven control processes on task-switching performance in middle childhood. Moreover, we manipulate the level of stimulus interference to examine effects on both preparation and target-driven control.

**Figure 1:**
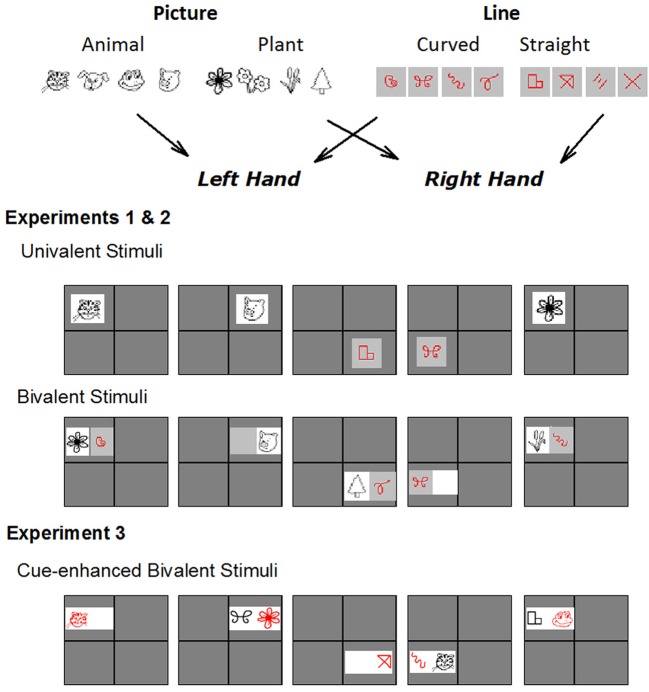
**Alternating runs paradigm is shown, illustrating tasks used for each experiment**. Participants switched between picture (animal or plant?) and line (curved or straight?) tasks using an AABB sequence. Response keys were bivalently mapped to the two tasks. In Experiments 1 and 2, the univalent condition included an exemplar only from the relevant task on each trial. The bivalent condition included exemplars from both tasks on two thirds of trials. Half of these were mapped to a response from the same hand (bivalent congruent) and the other half to responses from different hands (bivalent incongruent). In Experiment 3, all stimuli were bivalent but the relevant stimulus dimension was highlighted in red. See Methods for more detail.

In the alternating runs paradigm, the tasks alternate predictably in an AABB design. Thus, the identity of the currently relevant task is cued by trial position in the AABB sequence. That is, having completed two trials on Task A, the participant knows that they will switch to Task B for the following two trials, even before the appearance of the next stimulus. If the interval between their response to the preceding trial and the appearance of the next stimulus is long enough that allows participants to prepare in anticipation of the change in task (Rogers and Monsell, 1995). In addition, in most alternating runs paradigms, the spatial location of the stimulus itself also provides valid information of the task to be performed, as AABB sequences are mapped on a 2 × 2 matrix (see Figure 1). This contrasts with cued-trials designs where each trial consists of a cue-stimulus sequence and trial transition is randomized (Meiran et al., 2000), so that the participant does not know whether the upcoming trial will require a switch or repeat in task until the onset of the cue (for a critical evaluation of both cued-trials and alternating runs paradigms, see Altmann, 2007).

Cued-trials and alternating-runs paradigms have been shown to tap into distinct preparation processes. For instance, individuals who show intact advance preparation in cued task-switching may show impaired preparation when the task relies more heavily on internal monitoring. For example, in people with schizophrenia, ERP components associated with advance preparation are disrupted in an alternating runs paradigm (with an identical paradigm as that used in Experiment 2; see Karayanidis et al., 2006), but not a cued-trials paradigm (Jamadar et al., 2010a), indicative of specific difficulty in task preparation using internally generated cues (see also Werheid et al., 2007). Comparing the present outcomes with those in the young children from Manzi et al. (2011) will allow us to examine preparation with internal vs. external cues in young children.

We present three experiments that use the same base paradigm but differ in the degree of interference between task sets. In all experiments, participants are required to classify simple line drawings as representing either an animal or a plant (picture task) and sets of lines as either straight or curved (line task; Figure 1). The design was modeled after that of Rogers and Monsell (1995), but the letter and digit classification tasks were replaced with tasks that were more likely to be familiar to children as young as 6 years^2^. In order to ensure that these tasks produced the same outcomes as the original tasks, in Experiment 1, we tested the new stimulus sets in a group of young adults who completed a design identical to the one we have used previously (Karayanidis et al., 2003) including both univalent and bivalent stimulus sets (see below and Figure 1). In Experiment 2, we examined children's task-switching performance using a univalent version of the stimulus sets, so as to minimize stimulus-level interference from the irrelevant task set. In Experiment 3, both adults and children completed a variation of the above paradigm that included bivalent stimuli. However, the stimulus itself included a clear and unambiguous cue indicating the relevant task (Figure 1). This allowed us to examine age differences in stimulus-level interference without increasing task ambiguity. Although experiments differed in whether the stimuli were univalent or bivalent, responses were bivalent in all three experiments with the same two response keys mapped to both tasks (Figure 1).

## Experiment 1: task-switching with univalent and bivalent stimuli—task validation in young adults

We first confirmed that these new child-friendly tasks produce findings consistent with those previously obtained with the alternating runs paradigm (Rogers and Monsell, 1995; Karayanidis et al., 2003). Adults completed the task with both univalent and bivalent stimulus sets. In univalent conditions, each trial included an exemplar that was unambiguously mapped to the relevant task (Figure 1). In the bivalent conditions, in addition to the exemplar from the relevant task, 2/3 of trials included a second exemplar from the irrelevant stimulus set. So, for both univalent and bivalent stimulus conditions, the position of the stimulus in the 2 × 2 grid as well as its sequence in a set of trials (i.e., AABB) determined which task was relevant for that trial. However, in the bivalent condition, on most trials, the stimulus itself contained features from both tasks, and was therefore highly prone to stimulus-level interference. We examined ERP measures of anticipatory and stimulus-driven contributions to task-switching performance. Based on previous studies with the same design but different stimulus sets, we expected a significant RT switch cost that would reduce with increasing RSI, and that response-locked and stimulus-locked ERP waveforms will replicate established effects of task-switching.

### Methods

#### Participants

Eighteen undergraduate students (21.4 ± 6.2 years, 15 women) participated for course credit. Participants were naïve to task-switching paradigms and gave written informed consent.

#### Stimuli and tasks

Picture and line classification tasks were developed using highly representational stimuli (Figure 1). Stimuli for the picture task consisted of line drawings of four animals and four plants drawn in black on a white tile. Stimuli for the line task consisted of four line drawings using straight lines and four line drawings using curved lines drawn in white on a purple tile. All stimuli were presented against a black background. Response keys were bivalently mapped to the two tasks (Figure 1). Participants responded animal or plant for the picture task and straight or curved for the line task using their left or right index finger. The hand assigned to each button was counterbalanced across participants. Each stimulus remained on the screen until the participant responded or until 5 s had elapsed. Participants received immediate auditory feedback after every incorrect response and the subsequent stimulus was delayed by 1500 ms. After each run, performance feedback was given, including mean response time and number of errors.

On each trial, a stimulus was displayed in one of four boxes of a 2 × 2 matrix that was continuously displayed on the screen (1 m viewing distance). The picture and the line task were assigned to upper and lower boxes for half the participants and to right and left boxes for the other half. This ensured that, for half the participants, switch trials occurred on a vertical eye shift, and for the other half, switch trials occurred on a horizontal eye shift. As the display proceeded in a predictable clockwise manner, the position of the current stimulus provided a spatial cue as to the task active on the current trial as well as a valid task cue for the next trial (Figure 1).

In the univalent stimulus^3^ condition, the stimulus consisted of a single tile from the relevant task set. In the bivalent stimulus condition, the stimulus consisted of two tiles: one tile was selected from the relevant task set (Figure 1). The second tile was empty on one third of trials. On the remaining trials, the second tile was selected from the irrelevant task set and was mapped to either a same response hand as the relevant stimulus (congruent S-R mapping for relevant and irrelevant tile; 50%) or the opposite hand (incongruent S-R mapping for relevant and irrelevant tile; 50%).

#### Procedure

Participants completed two sessions scheduled about 1 week apart. Session one included practice with each task alone and with switching between tasks. Session two included further task practice, preparation for electroencephalogram (EEG) recording and experimental testing. All participants completed at least 500 practice trials. S-R and response-hand mappings were continuously displayed during practice, but were removed during testing. Testing consisted of six blocks of three runs (100 trials per run), including four blocks of the bivalent stimulus condition, one at each level of RSI (B150, B300, B600, B1200), and two blocks of the univalent stimulus condition at the two extreme RSIs (U150, U1200). This design replicated that used previously with letter and number tasks (Rogers and Monsell, 1995; Karayanidis et al., 2003). Block order was counterbalanced across subjects using a Latin square design. Participants were instructed to respond as quickly as possible while maintaining a high level of accuracy. Prior to each block, participants were informed whether the stimuli would be presented slowly (RSI 600 ms and 1200 ms) or quickly (RSI 150 ms and 300 ms) and encouraged to use the RSI to prepare for the next trial. The recording session took approximately 40 min.

#### Behavioral data analysis

The first four trials of each run, trials associated with an incorrect response or following an incorrect response, and trials associated with a response outside a 200–3000 ms time window were excluded from further analyses^4^. RT and arcsine transformed proportion error data (to account for the non-normal distribution) were analyzed, but percentage error is shown in figures.

Mean RT and error scores were analyzed separately for univalent and bivalent stimulus conditions. The univalent condition was analyzed using a 2 RSI (150, 1200) × 2 task (picture, line) × 2 trial type (switch, repeat) ANOVA. Residual RT switch cost was tested in long univalent condition (U1200) using a 2 task (picture, line) × 2 trial type (switch, repeat) ANOVA. The bivalent condition was analyzed using a 4 RSI (150, 300, 600, 1200) × 2 task (picture, line) × 2 trial type (switch, repeat) repeated measures ANOVA averaged across irrelevant character type^5^. Significant effects of RSI for the bivalent condition were examined with pairwise contrasts between adjacent RSI values. Bonferroni adjustment of type 1 error rate at α = 0.05 was used for all contrasts.

#### ERP recording and analysis

Electroencephalogram (EEG) was recorded continuously from 12 scalp electrodes (Fz, Cz, Pz, Oz, F3, C3, P3, T5, F4, C4, P4, T6) using an electrode cap (Electro-cap International) and linked mastoids reference. Vertical and horizontal electro-oculogram (EOG) were recorded from electrodes attached to the supra-orbital and infra-orbital ridges of the left eye and the outer canthi of each eye, respectively. EEG and EOG were continuously sampled at 500 Hz/channel using NeuroScan software and amplified (× 5000 for EOG and frontal channels; × 20 000 for other EEG channels) using a Grass Neurodata system (Model 12) with a bandpass of 0.01–30 Hz (−6 dB down).

Vertical eye movement artifact was corrected (Semlitsch et al., 1986) and sections with movement artifact or channel saturation were excluded from the continuous EEG files. Response-locked and stimulus-locked ERP epochs (1400 ms around response/stimulus onset; 200 ms pre-onset interval) were averaged separately for switch and repeat trials, resulting in 12 response-locked and 12 stimulus-locked ERP average waveforms for each participant at each site. Response-locked epochs within each condition were averaged separately depending on whether the following stimulus (i.e., trial *n* + 1) would require a switch or repeat trial. Baseline was corrected over −50–50 ms to avoid effects of large pre-baseline shifts in some conditions (Karayanidis et al., 2003).

Difference waveforms were derived by subtracting the average ERP switch waveform from the average ERP repeat waveform for each condition, trial type and participant. Difference waveforms were analyzed using point-by-point *t*-tests to establish points of significant positive deviation from baseline over 50–700 ms for response-locked waveforms (for B1200 and U1200 analyses were extended to 1200 ms to cover the entire RSI) and negative deviation from baseline over 50–1000 ms for stimulus-locked waveforms. The Guthrie and Buchwald (1991) procedure was used to control for Type 1 error at α = 0.05 using an autocorrelation coefficient of 0.9. Only effects significant by these criteria are reported. These analyses were used to define mean amplitude windows to examine variation in switch-related differences across RSI and group.

For both behavioral and ERP analyses, when appropriate, degrees of freedom were adjusted using Greenhouse–Geisser correction for the violation of the assumption of sphericity (Vasey and Thayer, 1987). For simplicity, figures show waveforms at Pz where effects tended to be largest, but results from four midline electrodes are shown in Table 1.

**Table 1:** **Areas of significant deviation from baseline for the switch-positivity in response-locked ERPs and the switch-negativity in stimulus-locked ERPs for Experiments 1 and 2 [*p* < 0.05 after correction using (Guthrie and Buchwald, 1991) criteria, see Methods for more details]**.

**Adults**	**B150**	**B300**	**B600**	**B1200**	**U150**	**U1200**	**Children**	**U150**	**U300**	**U600**	**U1200**
**RESPONSE-LOCKED ERP SWITCH-POSITIVITY: SWITCH > REPEAT**											
Fz				402–460	336–396		Fz	96–120	194–228		
								168–286	298–448		
								380–540	592–636		
Cz	166–210	276–472	312–488		82–108	298–342	Cz	52–304	154–700	98–142	345–460
	256–470				194–452			370–700		158–188	648–696
										432–464	
Pz	268–464	298–566	356–492		74–106	312–342	Pz	42–100	148–630	412–490	406–470
			520–590		190–226			136–700		610–700	650–700
					276–490						
Oz	296–440	334–402			160–184		Oz	136–158	134–258	122–154	426–462
		446–512			194–218			406–506		410–484	
					318–434					610–700	
**STIMULUS-LOCKED ERP SWITCH-NEGATIVITY: SWITCH < REPEAT**											
Fz	364–470	312–470	332–384	318–344		362–454	Fz	404–432		380–472	
			412–438	362–456				650–676			
			454–492					820–862			
Cz	348–510	284–566	134–158	128–154	342–476	280–456	Cz		154–198	252–506	
		924–956	218–542	256–486						818–850	
		996–1000	838–1000	496–524						934–960	
Pz	438–472	296–594	70–1000	256–558	738–1000	284–458	Pz		436–498	294–348	
	844–1000	750–100		868–984		776–962				408–490	
Oz	798–1000	276–594	66–1200	262–470	744–1000	288–436	Oz		462–512	434–486	
		634–656		860–912		748–922				656–688	
		692–1000		944–1000							

### Results

#### Behavioral data

**Figure 2:**
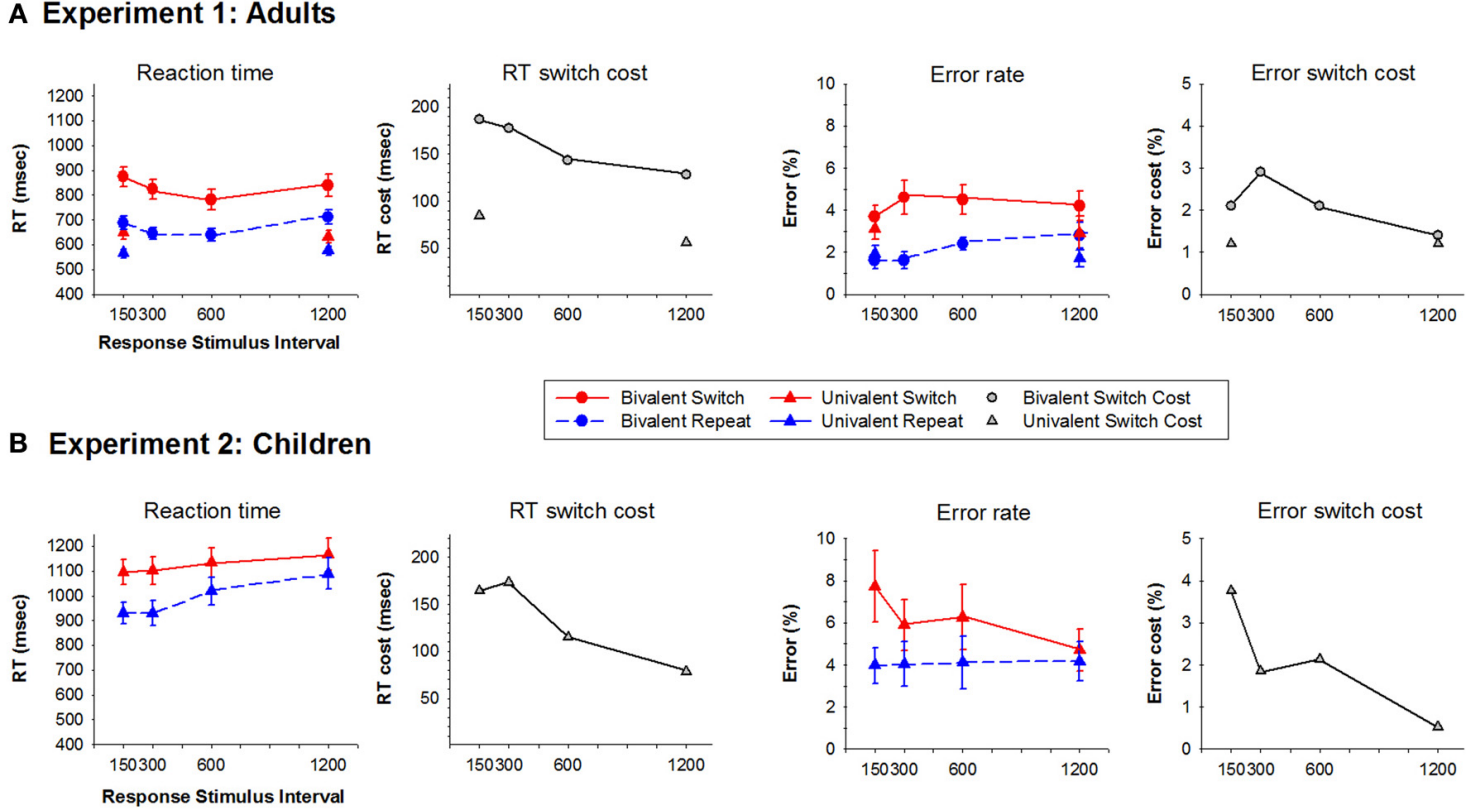
**Average reaction time and error rate for switch and repeat trials and their switch cost at each RTI condition for Experiments 1 (Adults) (A) and 2 (Children) (B)**. Circles represent bivalent stimulus blocks and upward triangles represent univalent stimulus blocks.

For the bivalent condition, mean RT was longer for the picture task than the line task [*F*_(1, 17)_ = 20.6, *p* < 0.001], however, task did not interact with RSI or trial type. RT varied significantly with RSI [*F*_(3, 51)_ = 9.2, *p* < 0.001], *reducing* as RSI increased from 150 to 300 ms [*F*_(1, 17)_ = 10.3, *p* = 0.005] and *increasing* as RSI increased from 600 to 1200 ms [*F*_(1, 17)_ = 21.8, *p* < 0.001; Figure 2A]. There was a significant RT switch cost [*F*_(1, 17)_ = 51.2, *p* < 0.001] that reduced with increasing RSI [*F*_(3, 51)_ = 9.7, *p* < 0.001] from 187 ms at B150–128 ms at B1200. This reduction in RT switch cost resulted from a 41 ms reduction in switch trial RT from B300–B600 and a 73 ms *increase* in repeat trial RT from B600–B1200.

The univalent condition resulted in a similar pattern of effects. RT was longer for the picture than the line task [*F*_(1, 17)_ = 18.4, *p* < 0.001]. A significant RT switch cost [*F*_(1, 17)_ = 22.3, *p* < 0.001] reduced with increasing RSI [*F*_(1, 17)_ = 12.8, *p* = 0.002], but a significant 50 ms residual RT switch cost remained at U1200 ms [*F*_(1, 17)_ = 15.7, *p* < 0.001].

Error rate was very low (1.4–5.6% across conditions). Both bivalent and univalent conditions had a significant switch cost [*F*_(1, 17)_ = 22.3, *p* < 0.001, *F*_(1, 17)_ = 21.9, *p* < 0.001]. Reduction in error switch cost with increasing RSI was only evident for the bivalent condition where it was marginally significant (*p* < 0.10).

#### Response-locked ERP data

**Figure 3:**
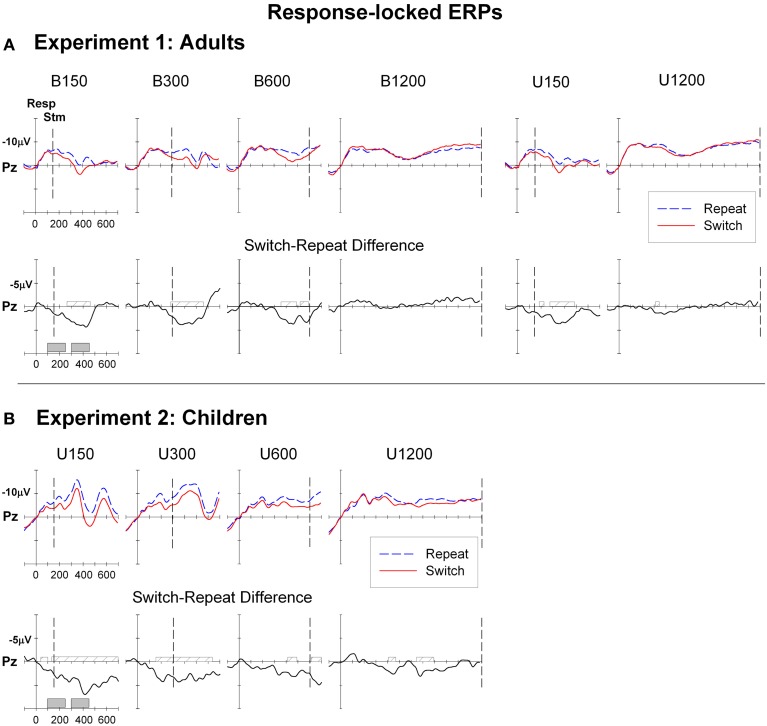
**Response-locked ERPs for switch and repeat trials and (switch—repeat) difference waveforms at Pz for (A): Experiment 1 (Adults) and (B): Experiment 2 (Children)**. Filled gray bars on timeline indicate epochs used for mean amplitude measures. Checked bars on x-axis of difference waveforms indicate regions of significant positive deviation from baseline. Negative is plotted up.

ERP waveforms averaged from the onset of the response to the preceding stimulus and extending across the RSI are shown at Pz in Figure 3A for bivalent and univalent conditions. Both conditions and all RSIs showed a post-response negative shift that was larger parietally and peaked 100–200 ms post-response. After 200 ms, the morphology of the response-locked ERPs varied depending on RSI length. In the shorter RSI conditions (B150, B300, U150), the negativity overlapped with ERPs associated with stimulus processing. For B600, a sustained negativity remained centroparietally until stimulus onset, whereas for the long RSI conditions (B1200, U1200), a second late pre-stimulus negativity emerged centroparietally.

For the bivalent condition, differences between ERPs in anticipation of a switch or repeat trial were evident across all RSI levels (Table 1) but were more pronounced at central and parietal sites and for the three shorter RSIs. Difference waveforms (Figure 3A, lower panel) showed a broad centroparietal positivity reflecting the build-up of a positive shift for switch relative to repeat trials at the two shorter RSIs. This positivity significantly deviated from baseline at or before stimulus onset [e.g., earliest deviation at 160 ms for B150 (Cz) and at 276 ms for B300 (Pz)] and extended to ~500–550 ms post-response (Table 1). At B600, this differential switch-positivity emerged first centrally at 312 ms and extended parietally to stimulus onset (590 ms). At the longest RSI (B1200), there was a small switch dip that was not significant parietally, but that deviated from baseline over 402–460 ms frontally (Table 1). The univalent stimulus blocks showed a similar differential switch-positivity to that seen in their respective bivalent blocks (Figure 3A).

#### Mean amplitude analyses

**Figure 4:**
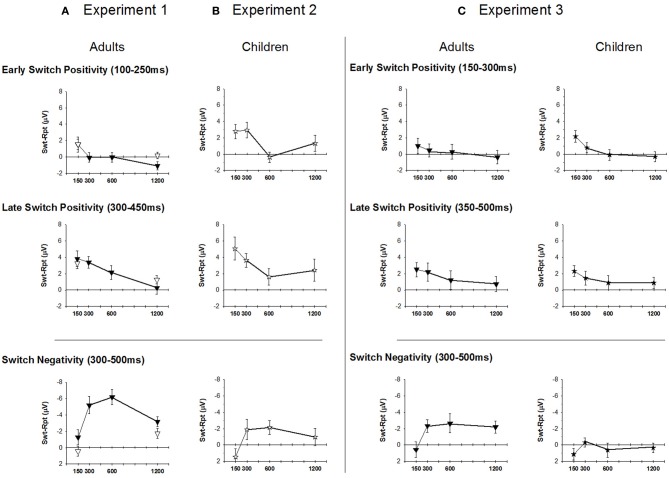
**Mean amplitude of early and late switch-positivity from response-locked ERP waveforms (top and middle panels) and switch-negativity from stimulus-locked ERP waveforms (bottom panel)**. **(A)**: Experiment 1 (Adults), **(B)**: Experiment 2 (Children), **(C)** Experiment 3 (Adults and Children). Downward triangle: Adults; Star: Children.

Two mean amplitude windows were defined based on the results of the point-by-point deviation from baseline. The first window (100–250 ms) targeted the early switch-positivity most clearly evident centroparietally at short RSIs. The second window (300–450 ms) captured the interval of maximal switch-positivity. These were analyzed using a 4 RSI × 4 midline electrode analysis to examine effects of RSI and a 2 condition × 2 RSI × 4 electrode analysis to examine whether RSI effects varied across univalent and bivalent conditions. Mean amplitude measures at Pz are shown in Figure 4A.

#### Early switch-positivity (100–250 ms)

In the bivalent condition, there was a significant decline in amplitude with increasing RSI [*F*_(3, 51)_ = 4.5, *p* = 0.014], reflecting the centroparietal switch-positivity for B150 being replaced by a fronto-central switch-negativity in all other conditions [B150 vs. later: *F*_(1, 17)_ = 7.5, *p* = 0.014; Table 1]. Early switch-positivity amplitude did not differ between univalent and bivalent conditions.

#### Late switch-positivity (300–450 ms)

In the bivalent condition, the late switch-positivity was larger centroparietally [*F*_(3, 51)_ = 5.7, *p* = 0.009] and reduced with increasing RSI, especially across central to occipital sites [RSI: *F*_(3, 51)_ = 4.4, *p* = 0.013; RSI × Elec: *F*_(9, 153)_ = 4.7, *p* = 0.003]. Late switch-positivity amplitude did not differ between univalent and bivalent conditions (Table 1).

#### Stimulus-locked ERP data

**Figure 5:**
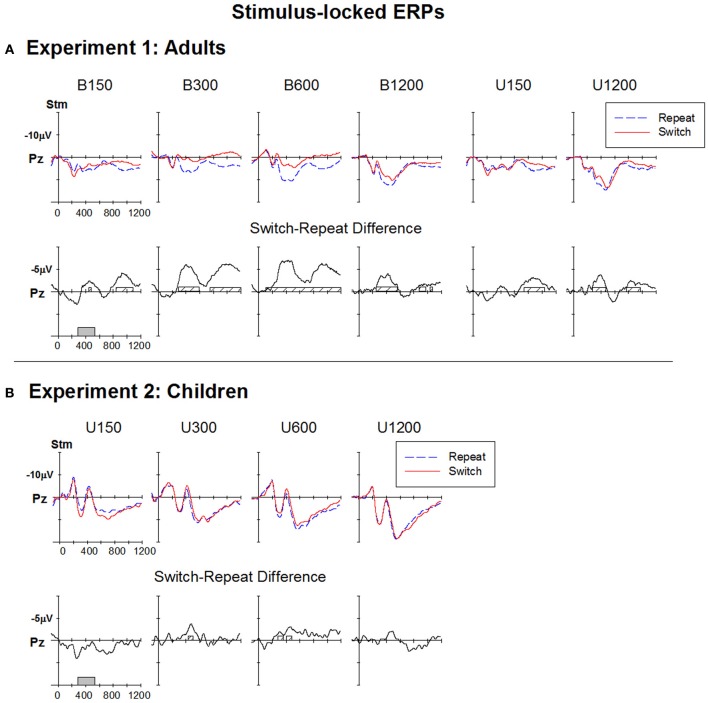
**Stimulus-locked ERPs for switch and repeat trials and (switch—repeat) difference waveforms at Pz for (A): Experiment 1 (Adults) and (B): Experiment 2 (Children)**. Filled gray bars on timeline indicate epochs used for mean amplitude measures. Checked bars on x-axis of difference waveforms indicate regions of significant positive deviation from baseline. Negative is plotted up.

Stimulus-locked waveforms for switch and repeat trials at each RSI are presented in Figure 5A. All conditions showed a centroparietal late positive component (LPC) over 300–600 ms which is not clearly evident at the shortest RSI conditions (B150, U150) which have substantial temporal overlap between response-locked and stimulus-locked waveforms (see Figures 3A, 5A). Note that, for these short RSI blocks, response- and stimulus-locked waveforms are identical with the exception that the baseline has shifted 150 ms to the right in the stimulus-locked ERP averages.

Differential processing of switch and repeat stimuli was evident as reduced LPC for switch as compared to repeat trials over 300–600 ms (Figure 5A; Table 1). Switch and repeat ERPs differentiated as early as 70 ms and spread over parietal P2 and N2 before peaking ~400 ms. The LPC was followed by a later centroparietal positivity (800 ms+) that was evident only for repeat trials.

#### Mean amplitude analyses

The differential switch-negativity was measured using mean amplitude over 300–500 ms at Cz and Pz where the effect was largest (Figure 4A, lower panel). For bivalent stimuli, the differential switch-negativity showed a large effect of RSI [*F*_(3, 51)_ = 5.3, *p* = 0.006], being larger at both B300 and B600 as compared to their neighboring interval [*F*_(1, 17)_ = 11.5, *p* = 0.003 and *F*_(1, 17)_ = 7.31, *p* = 0.015, respectively]. Switch-negativity amplitude did not differ significantly between bivalent and univalent conditions (*F* < 1.5).

### Discussion

These results closely replicate both behavioral and ERP findings with the classic Rogers and Monsell tasks (Karayanidis et al., 2003). Young adults showed a reduction in RT switch cost with increasing RSI for the bivalent stimulus condition as well as longer RT and larger RT switch cost for bivalent as compared to univalent conditions. For bivalent stimuli, reduction in RT switch cost resulted from a larger decrease in RT for switch than repeat trials as RSI increased from 150 to 600 ms, and a smaller increase in RT for switch than repeat trials as RSI increased further to 1200 ms. Error rate showed a small switch cost that was not reliably affected by RSI.

Response-locked ERPs showed a large switch-positivity that emerged earlier for short RSIs. At short RSIs, this switch-positivity extended beyond stimulus onset, overlapping several early and later ERP components associated with stimulus processing. At long RSIs, the switch-positivity emerged after 300 ms and was restricted within the RSI. Stimulus-locked ERPs produced a large centroparietal switch-negativity that peaked around 400 ms post-stimulus onset. This is consistent with the *differential switch-negativity* (Karayanidis et al., 2003, 2006) or reduction in LPC amplitude (e.g., Kieffaber and Hetrick, 2005; Astle and Scerif, 2009).

## Experiment 2—task-switching with univalent stimuli in middle childhood

Adults showed significant differences in switch cost between univalent and bivalent stimuli. Moreover, pilot testing suggested that the full six blocks made the testing session too long for younger children, producing fatigue-related performance decline and movement artifact. Hence, children were tested on four task blocks with univalent stimuli only; one at each RSI level.

Based on earlier studies using the alternating runs paradigm (Huizinga et al., 2006; Kray et al., 2008), we expected that children would show larger RT switch cost and greater reduction in RT switch cost with increasing preparation interval as compared to young adults. If this pattern is due to less efficient preparation in anticipation of a switch trial, children will show a smaller or later switch-positivity. If it is related to greater stimulus-level interference for switch trials, children will have a larger switch-negativity.

### Methods

The methods are identical to Experiment 1, with a few exceptions as noted below.

#### Participants

Sixteen children were recruited from two mainstream primary schools (9.46 ± 1.97 year, range = 6–12 year; 11 girls) and were naïve to task-switching paradigms. Mean IQ was 112.7 ± 7.6 using the Kaufman Brief Intelligence Test (K-BIT; Kaufman, 1990). Children and their parents gave written informed consent.

#### Stimuli and tasks

Children completed only the univalent stimulus condition at four levels of RSI presented in separate blocks (3 runs of 100 trials per block; U150, U300, U600, U1200).

#### Data analysis

Mean RT and error scores were analyzed using a 4 RSI (150, 300, 600, 1200) × 2 task (picture, line) × 2 trial type (switch, repeat) repeated measures ANOVA. Response-locked and stimulus-locked ERP epochs were averaged separately for switch and repeat trials at each RSI, resulting in 8 response-locked and 8 stimulus-locked average waveforms (4 RSI conditions × 2 trial type) at each site for each child.

### Results

#### Behavioral data

Figure 2B shows RT and error scores for switch and repeat trials, as well as their switch cost. As with the adult group, the picture task resulted in longer RT than the line task [*F*_(1, 15)_ = 8.5, *p* = 0.01], and switch trials produced longer RT than repeat trials [*F*_(1, 15)_ = 80.0, *p* < 0.001]. RT switch cost reduced with increasing RSI [*F*_(3, 45)_ = 9.9, *p* < 0.001] and, as with adults, this effect was largest as RSI increased from 300 to 600 ms [*F*_(1, 15)_ = 12, *p* = 0.004]. However, in children, RT *increased* across the entire RSI range [*F*_(3, 45)_ = 7.2, *p* < 0.001]. This effect was highly significant on repeat trials [*F*_(3, 45)_ = 10.5, *p* < 0.001] but only marginally significant for switch trials [*F*_(3, 45)_ = 2.3, *p* = 0.093]. A significant residual RT switch cost of over 70 ms remained at the longest RSI [*F*_(1, 15)_ = 33.4, *p* < 0.001]. Error rate was overall higher for switch than repeat trials [*F*_(1, 15)_ = 11.5, *p* = 0.004], but no other effects were significant.

#### Response-locked ERP data

Figure 3B shows that ERPs had a broadly similar morphology for children as for adults (Figure 3A), although amplitude was larger across the board for children (quantitative comparison of adults and children is presented in Supplementary Material). Like adults, children showed a broad negative-going waveform with large differentiation between switch and repeat waveforms, especially at short RSIs, but with very early emergence of the switch-positivity (Figure 3B). At U150, the switch-positivity significantly deviated from baseline from the beginning of the analysis epoch (50 ms). Such very early effects need to be interpreted with caution, given the sharp pre-response negative shift for both repeat and switch waveforms and the use of a ±50 ms baseline. Nevertheless, in children, an early differential switch positivity was evident even at the frontal site (96–120 ms; Table 1) despite a flat pre-response baseline and no pre-response differentiation of switch and repeat ERPs. For both U150 and U300 conditions, a single switch-positivity extended parietally across most of the 700 ms analysis epoch, whereas frontocentrally results are more compatible with two positivities, one around 100–300 ms and a second from 350 to beyond 700 ms (Table 1).

The switch-positivity was smaller and less widespread for longer RSI conditions. For U600, although the difference waveform consistently hovered below the baseline, there were only small areas of significant deviation from baseline at central, parietal and occipital sites extending from as early as 98 ms post-response to beyond stimulus onset (Table 1). U1200 showed a switch-positivity emerging at 350 ms followed by a second positivity over 650–850 ms.

#### Stimulus-locked ERP data

Children showed a similar pattern of stimulus-locked ERPs as adults, but as often reported, these components were more widespread and larger (Figure 5B; Supplementary Material). However, children showed minimal and temporally restricted differentiation between switch and repeat trial types (Table 1). For U300 and U600, a small switch-negativity was significant posteriorly only around 400–500 ms. No significant centroparietal switch-negativity was found at the two extreme RSI values.

### Discussion

Given differences in stimulus conditions between Experiments 1 and 2, we cannot directly statistically compare performance of adults and children. However, we discuss the performance of children in Experiment 2 relative to that of adults in Experiment 1, taking into consideration that adults completed all four RSI conditions with bivalent stimuli and only the two extreme RSI conditions with univalent stimuli. For the interested reader, direct comparison of adults from Experiment 1 and children from Experiment 2 for the two common RSI conditions with univalent stimuli (U150 and U1200) is provided in Supplementary Materials.

#### Behavioral data

On an alternating-runs task-switching paradigm with univalent stimuli, children showed a significant RT switch cost (Huizinga et al., 2006; Kray et al., 2008) that reduced with increasing RSI (Cepeda et al., 2001). Surprisingly, despite the fact that the children ranged in age from 6 to 12 years, there was no variation in switch cost with increasing age (*p* > 0.10). While this may be partly due to the relatively small sample size, the finding is compatible with previous studies showing no change in RT switch cost across a similar age range (Kray et al., 2004; Reimers and Maylor, 2005; Karbach and Kray, 2007).

While the overall pattern of RT switch cost that reduces with increasing RSI was evident for both adults (Experiment 1) and children (Experiment 2), in adults, the reduction in RT switch cost was primarily due to relatively faster RT on switch trials (Figure 2A), whereas in children it was due to slower RT on repeat trials, especially in longer RSI conditions (Figure 2B). On the basis of behavioral results alone, the mechanism underlying task-switching performance in children is not immediately evident. However, the different pattern of RSI effects on mean RT for switch and repeat stimuli in adults and children suggest differences in task strategy. In adults, the reduction in RT switch cost as RSI increased from 150 to 600 ms was mostly attributable to a larger reduction in RT for switch as compared to repeat trials (Figure 2A). There was no further reduction in RT or RT switch cost from 600 to 1200 ms RSI (Rogers and Monsell, 1995). In children, the reduction in RT switch cost as RSI increased from 150 to 300 to 600 ms appears to reflect different underlying processes. Figure 2B shows that, for both trial types, RT *increased* as RSI increased from 300 to 600 ms, and that this effect of RSI on RT was larger for repeat stimuli thereby resulting in a significant reduction in RT switch cost. This suggests that the benefit of repeating a task is reduced for children in long vs. short RSI conditions—in other words, the benefit of having just performed the currently relevant task dissipates faster in children than in adults. This is compatible with Cragg and Nation's (2009) finding that young children show smaller RT switch cost to high vs. low interference stimuli because of larger RT for repeat trials. These findings, in combination with an overall increase in error rate for children, suggest strategic differences in task-switching performance, even on a simple univalent stimulus version of the alternating runs task-switching paradigm that clearly differentiates between the two tasks on the basis of sequence, location, and stimulus set.

#### Response-locked ERPs

Children showed a similar pattern to adults with some exceptions. A large differential switch-positivity was evident across most RSI conditions, emerging as early as 50 ms post-response for short RSI conditions and extending across the analysis window and across midline sites. So, the switch-positivity emerged earlier in children than in adults, but like adults, children showed a maximum effect in the 300–450 ms window. These findings are partly consistent with Manzi et al. (2011) who used a cued-trials paradigm with bivalent stimuli and also found no age difference in peak amplitude of the switch-positivity. However, in contrast to our findings, their switch-positivity emerged later and had a shorter duration for children than adults.

#### Stimulus-locked ERPs

Children showed very large stimulus-locked ERP components but little differentiation between switch and repeat trials. The switch-negativity is superimposed upon the P3b that is known to decrease in amplitude with increased effort/conflict. Therefore, this effect could be partly due to the fact that univalent stimuli produce less post-stimulus interference and more efficient processing of switch trials, i.e., switch trials are processed as efficiently as repeat trials. However, this would suggest that the amplitude of the switch-negativity should be smaller for univalent as compared to bivalent stimulus conditions in adults, which was not the case in these data. The smaller switch-negativity in children as compared to adults may indicate either that children are processing switch trials more like repeat trials or that they are processing repeat trials more like switch trials. The former would suggest that children experience less post-stimulus interference on switch trials than adults, whereas the latter would suggest that children experience greater post-stimulus interference than adults, and disproportionately so on repeat trials. Both alternatives are compatible with no differential increase in RT switch cost for children. However, the findings that children responded more slowly overall, progressively increased RT with RSI, especially for repeat trials, and showed larger reduction in RT switch cost with increasing RSI are more compatible with greater post-stimulus interference especially on repeat trials (Cepeda et al., 2001).

### Conclusion

With univalent stimuli, children showed slower RT, larger RT switch cost and greater reduction in RT switch cost than seen in Experiment 1 with adults. The switch-positivity emerged early and was very widespread in children, suggesting intact and perhaps greater activation of anticipatory processes. Importantly, children showed little evidence of a switch-negativity in the stimulus-locked waveforms. Together with increased RT switch cost and increasing repeat trial RT over RSI, this suggests that, despite greater activation of anticipatory reconfiguration processes during the preparatory period, children experience more post-stimulus interference during task implementation, and that this differentially affects processing of repeat trials, resulting in little post-stimulus differentiation between switch and repeat trials.

## Experiment 3—task-switching with bivalent stimuli in adulthood and childhood

Experiment 2 showed that children have difficulty with task implementation, even with univalent stimuli and long preparation intervals. In Experiment 3, we examine this effect further by using bivalent stimuli. We expected that children would show greater disruption of task-switching performance, and that this would be more pronounced on stimulus-locked processing of repeat trials. This paradigm included only bivalent stimuli, but each stimulus included an additional redundant cue that unambiguously marked the relevant stimulus dimension. Specifically, as shown in Figure 1, on each trial, the relevant task dimension was outlined in red (e.g., if picture task was relevant, animals and plants were drawn in red ink), whereas the irrelevant was outlined in black (e.g., in the above example, straight and wavy lines were drawn in black ink). This condition allowed us to examine the effect of stimulus-driven interference from the irrelevant stimulus dimension without any ambiguity about which task was relevant for that trial. We reasoned that this condition would involve greater stimulus-driven interference than the univalent task that children completed in Experiment 2 and but less than the bivalent task that adults completed in Experiment 1.

### Methods

#### Participants

Sixteen undergraduate students (6 male, mean age = 22.6 ± 3.8 years) and 28 children from two local primary schools (15 male, mean age 9.1 ± 2.2 years; 5.6–12.9 years) without prior exposure to similar paradigms, participated in this study. Children had an average IQ of 110 ± 10.1 on the K-BIT (Kaufman, 1990). Children and their parents as well as adult participants gave written consent.

#### Stimuli and task

The stimuli and tasks were similar to those used in Experiment 1 with the following modifications. Firstly, all groups received only the bivalent stimulus condition, with blocked RSI of 150, 300, 600, and 1200 ms. Secondly, all stimuli were presented against a white background. Finally, on each trial, the relevant task was signaled not only by the position of the stimulus in the 2 × 2 matrix (e.g., top vs. bottom boxes) and the sequence of preceding trials (i.e., AABB), but also by the color of the stimulus (Figure 1). The relevant stimulus was always presented in red and the irrelevant stimulus was always presented in black, providing an extra clue as to which task was relevant on any given trial. All remaining parameters were identical to those used in Experiment 1.

#### Behavioral and ERP data

**Figure 6:**
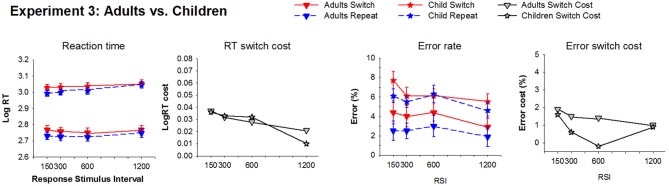
**Average reaction time and error rate for switch and repeat trials and their switch cost at each RSI condition for Experiment 3**. Downward triangle: Adults; Star: Children.

Behavioral and ERP data were recorded and processed as reported in Experiment 1. Mean RT and arcsine transformed error rate were analyzed using a 4 RSI (150, 300, 600, 1200 ms) × 2 task (picture, line) × 2 trial type (switch, repeat) mixed design ANOVA for adult and children separately and group was added as a between subjects factor to compare adults and children. Significant effects of RSI were examined by comparing adjacent RSI values. Residual RT switch cost was tested in the 1200 ms RSI condition using a 2 task × 2 trial type ANOVA. Bonferroni adjustment of type 1 error rate was used for all contrasts. In order to directly compare RT effects across the two age groups, we used log-RT scores in all analyses (see Figure 6). However, all results were largely identical when using RT scores instead. We used the acronym EB (enhanced bivalent) to differentiate the current bivalent condition from the univalent (U) and bivalent (B) conditions in Experiments 1 and 2.

### Results

#### Behavioral data

***Adults***. RT was longer and error rate higher for the picture task compared to the line task [*F*_(1, 15)_ = 36.6, *p* < 0.001; *F*_(1, 15)_ = 8.1, *p* = 0.012], however, task did not interact with any other factor. As shown in Figure 6, there was a significant RT switch cost [*F*_(1, 15)_ = 62.9, *p* < 0.001] that showed a small (20 ms) but significant reduction with RSI [*F*_(3, 45)_ = 3.7, *p* = 0.027]. While switch trial RT did not vary significantly with increasing RSI (*p* > 0.12), the main effect of RSI was significant for repeat trials [*F*_(3, 45)_ = 5.6, *p* = 0.012] due to a 30 ms increase from EB600 to EB1200 [*F*_(1, 15)_ = 10.2, *p* < 0.006], consistent with the equivalent condition in Experiment 1. The 30 ms residual switch cost was statistically significant [*F*_(1, 15)_ = 26, *p* < 0.001]. Error rate reduced with increasing RSI [*F*_(1, 15)_ = 4.0, *p* < 0.022] and also showed a significant switch cost [*F*_(1, 15)_ = 10.6, *p* = 0.005] that did not reduce with RSI.

***Children***. As shown in Figure 6, children also showed a significant RT switch cost [*F*_(1, 27)_ = 17.4, *p* < 0.001] that reduces with increasing RSI [*F*_(3, 81)_ = 5.7, *p* = 0.002; 95 ms at EB150, 27 ms at EB1200] with a marginally significant residual RT switch cost [*F*_(1, 27)_ = 3.9, *p* = 0.058]. As in Experiment 2, the reduction in RT switch cost resulted from smaller increase in RT for switch [40 ms; *F*_(3, 81)_ = 3.8, *p* = 0.018] as compared to repeat trials [135 ms; *F*_(3, 81)_ = 11.9, *p* < 0.001] across the RSI range. Children also produced a significant error switch cost [*F*_(1, 27)_ = 9.6, *p* = 0.005], especially at short RSI [*F*_(3, 81)_= 6.6, *p* < 0.001], but there was no significant reduction in error switch cost with increasing RSI.

***Comparison between Adults and Children***. As expected, children were overall slower than adults [*F*_(1, 42)_ = 52, *p* < 0.001]. A significant group by RSI interaction [*F*_(3, 126)_ = 3.4, *p* = 0.021] reflected that RT increased more from EB150 to EB1200 in children as compared to adults [*F*_(1, 42)_ = 4.1, *p* = 0.05]. There was no age difference in RT switch cost or its reduction with increasing RSI. Children made overall more errors than adults [*F*_(1, 42)_ = 6.9, *p* = 0.012], but group did not interact with either RSI or trial type.

#### Response-locked ERP

**Figure 7:**
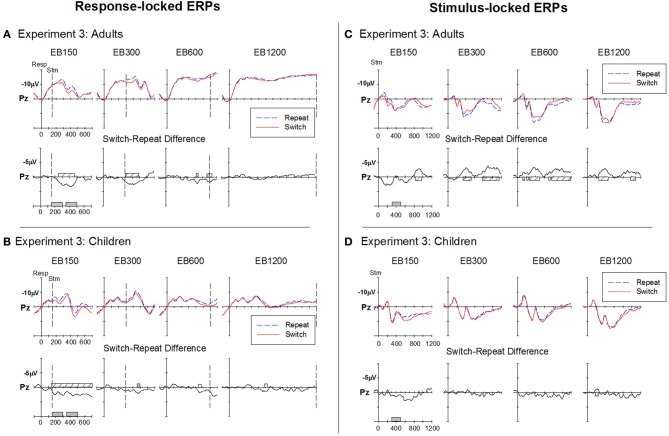
**Response-locked (A,B) and stimulus-locked (B,C) ERPs for switch and repeat trials and (switch—repeat) difference waveforms at Pz for Experiment 3**. Filled gray bars on timeline indicate epochs used for mean amplitude measures. Checked bars on x-axis of difference waveforms indicate regions of significant positive (left panel) or negative (right panel) deviation from baseline. Negative is plotted up.

Response-locked ERP waveforms for adults and children (Figures 7A,B) showed very similar morphology to that seen in Experiments 1 and 2.

***Adults***. A large centroparietal switch-positivity was more clearly evident for the two fast RSI conditions (i.e., EB150 and EB300), spreading from around 225–500 ms (Figure 7A). The switch-positivity emerged later (~300 ms) and was smaller and more widespread for EB600. The small positive shift around 300–400 ms for RSI-1200 did not reach statistical significance (see Table 2).

**Table 2:** **Areas of significant deviation from baseline for the switch-positivity in response-locked ERPs and the switch-negativity in stimulus-locked ERPs for Experiment 3 (*p*<0.05 after correction using (Guthrie and Buchwald, 1991) criteria, see Methods for more details)**.

**Adults**	**B150**	**B300**	**B600**	**B1200**	**Children**	**U150**	**U300**	**U600**	**U1200**
**RESPONSE-LOCKED ERP SWITCH-POSITIVITY: SWITCH > REPEAT**									
Fz	244–444	340–464	370–470		Fz	154–818	98–310		
Cz	224–472	296–488	306–476		Cz	50–100	100–312		152–178
			562–678			108–828	432–490		864–902
Pz	246–484	302–484	418–444		Pz	144–956	458–488	444–476	490–520
			566–634						
Oz	312–432	328–364			Oz				
**STIMULUS-LOCKED ERP SWITCH-NEGATIVITY: SWITCH < REPEAT**									
Fz		280–530	116–226	132–178	Fz		276–370		170–240
			280–424	284–450			474–872		
Cz		256–528	114–500	132–174	Cz				164–214
		842–1020	808–928	256–502					
				668–724					
				760–1000					
Pz	828–984	322–526	108–160	248–476	Pz				190–236
		764–1000	200–230	790–1000					
			258–504						
			706–744						
			768–1000						
Oz	828–992	850–1000	268–468	242–452	Oz				

***Children***. At the fastest RSI (i.e., EB150), children showed a large centro-parietal switch-positivity extending from as early as 50 ms to the end of the analysis period (Figure 7B). A smaller switch-positivity emerged for EB300 especially at the central site (Table 2). At longer RSIs, there was only a small switch-positivity at the parietal site that was temporally restricted.

***Comparison between Adults and Children***. Two mean amplitude windows were defined to capture the early onset of the switch-positivity (150–300 ms) seen especially in children and the later switch-positivity (350–500 ms) that was more prominent in adults (Figure 4C). These were analyzed separately using a 2 group × 4 RSI × 2 electrodes (Cz, Pz) where the positivity was largest. Both early and late switch-positivities reduced in amplitude with increasing RSI [*F*_(3, 126)_ = 4.8, *p* < 0.001; *F*_(3, 126)_ = 2.9, *p* = 0.046] but there was no effect of age group (*F* < 1.5).

#### Stimulus-locked ERP

***Adults***. Stimulus-locked ERPs showed a switch-negativity emerging as early as 100 ms post-stimulus parietally (see EB600) and extending to around 530 ms (Figure 7C). This was followed by a second negative shift for switch vs. repeat trials that extended beyond 1000 ms in most RSI conditions. At EB150, the switch-negativity was preceded by the switch-positivity that peaked after stimulus onset when the RSI was very brief.

***Children***. While stimulus-locked ERP waveforms showed a very similar morphology to that seen in Experiment 1, there was no evidence of a switch-negativity for any RSI condition (Figure 7D).

***Comparison between adults and children***. As in Experiments 1 and 2, switch-negativity amplitude was measured over 300–500 ms and analyzed using a 2 group × 4 RSI × 2 electrode mixed design ANOVA. The switch-negativity increased from EB150 to EB300 [*F*_(1, 42)_ = 10.6, *p* = 0.002], but remained consistent thereafter (Figure 4C, lower panel). It was significantly larger for adults across the range of RSI [*F*_(1, 42)_ = 17.2, *p* < 0.001].

### Discussion

Experiment 3 included a similar design as Experiment 2 with two important exceptions. Firstly, both adults and children were exposed to the same bivalent stimuli and secondly, although the stimuli were bivalent, an extra redundant cue was included to clearly indicate the relevant stimulus dimension and associated task on each trial. In Experiment 2, we found that children show post-stimulus interference even with univalent stimuli, especially for repeat stimuli. In this experiment, we expected that bivalent stimuli would produce greater performance disruption for children than adults.

Both children and adults showed a significant switch cost for error rate and RT. Neither the size of the RT switch cost nor the rate of reduction with increasing RSI differed between children and adults. However, the reduction in switch cost resulted largely from increasing RT for repeat trials, an effect most prominent in children (135 vs. 31 ms for children and adults, respectively). Both groups showed a switch-positivity that was largest at the short RSI condition and was less pronounced at longer RSIs. As in Experiment 2, children showed a large switch-positivity similar to that of adults (Manzi et al. (2011) but the switch-negativity was completely absent in children.

## Cross-experiment comparison: stimulus valence effects on task-switching

### Stimulus valence on task-switching in children

To examine switching with univalent vs. bivalent stimuli in children, we compared results from Experiment 2 (univalent stimuli) and 3 (bivalent stimuli). Children in Experiments 2 and 3 did not differ significantly in either age or IQ scores (*p* > 0.10). The two groups were compared using a 2 Experiment (Experiment 1: univalent, Experiment 2: bivalent) × RSI (4) × Trial Type (2) × Task (2) mixed design ANOVA. Although RT did not differ between the two experiments, RT switch cost was larger for univalent (133 ms for Experiment 1) than enhanced-cue bivalent stimuli [74 ms for Experiment 2; *F*_(1, 42)_ = 4.9, *p* = 0.032]. Mean RT for switch trials differed by only 17 ms, whereas repeat trial RT differed by 75 ms, suggesting that the reduction in RT switch cost with the more difficult bivalent stimuli in Experiment 3 resulted from a greater increase in repeat trial RT.

### Stimulus valence on task-switching in adults

Comparison of univalent stimuli (Experiment 1) and cue-enhanced bivalent stimuli (Experiment 3) at the two common RSI conditions (150 and 1200 ms) showed no significant experiment effect or interaction (*p* > 0.05). In contrast, there were significant performance differences between bivalent stimuli in Experiment 1 and cue-enhanced bivalent stimuli in Experiment 3. Specifically, when the relevant dimension of the bivalent stimulus was not highlighted (Experiment 1), adults responded more slowly [750 vs. 560 ms; *F*_(1, 32)_ = 26.7, *p* < 0.001], produced a larger RT switch cost [160 vs. 40 ms; *F*_(1, 32)_ = 24.8, *p* < 0.001], and a larger reduction in switch cost with increasing RSI [57 vs. 20 ms; *F*_(3, 96)_ = 3.7, *p* = 0.016] than when the relevant dimension was highlighted (Experiment 3). While the effect of Experiment was significant on both repeat and switch trial RT [*F*_(1, 32)_ = 18.9, *p* < 0.001; *F*_(1, 32)_ = 29.7, *p* < 0.001, respectively], repeat trials were approximately 130 ms slower whereas switch trials were more than 250 ms slower when the stimulus itself did not contain information indicating which task is relevant^6^.

### Conclusion

Adults performed equivalently for univalent stimuli in Experiment 1 and enhanced-cue bivalent stimuli in Experiment 2. This indicates that they effectively used the redundant cue to focus on the relevant task dimension and reduce stimulus-driven interference from the irrelevant stimulus dimension. In contrast, adults performed more poorly with bivalent stimuli when they did not include an explicit cue highlighting the relevant dimension (e.g., Experiment 1 vs. 3). This was due to a disproportionate increase in RT for switch as compared to repeat trials, suggesting that the irrelevant stimulus dimension interfered with task-set implementation on switch trials, even under highly prepared and practiced performance.

Rather unexpectedly, children produced a larger RT switch cost with the *univalent stimuli* (Experiment 2) than with the enhanced-cue bivalent stimuli (Experiment 3). In other words, they had a larger RT switch cost when the stimulus clearly and unambiguously indicated which task was relevant and included no information pertaining to the irrelevant task set (Experiment 2) than when the stimulus included exemplars from both task sets, but unambiguously cued the relevant dimension (Experiment 3). Importantly, the larger RT switch cost for univalent as compared to enhanced-cue bivalent stimuli resulted from a relative modulation of *repeat trial* RT. Specifically, children appear to deal with switch trials equivalently for both univalent and enhanced-cue bivalent stimuli, but to be differentially slowed for repeat trials with enhanced-cue bivalent stimuli. So, the presence of the exemplar from the irrelevant stimulus dimension in Experiment 3 appears to have disproportionately affected children's responding on repeat trials. This suggests that children found it harder to ignore irrelevant information on repeat trials in Experiment 3. The finding that the switch-negativity in children was small (Experiment 2) or absent (Experiment 3), is compatible with the argument that children encounter a greater level of post-stimulus interference on repeat trials, particularly in the presence of irrelevant information.

## General discussion

### Switch cost in middle childhood

When switching with either univalent or cue-enhanced bivalent stimuli, children showed a reduction in RT switch cost with increasing time for preparation (Cepeda et al., 2001). In response-locked ERPs, children showed a large and sustained switch-positivity with univalent stimuli (Experiment 2) but a much smaller switch-positivity with the cue-enhanced bivalent stimuli (Experiment 3), consistent with differential preparation for a switch trial, especially in the shorter RSI conditions Manzi et al. (2011). The reduced switch-positivity at longer preparation intervals in children may indicate either poor preparation to switch (e.g., children may have difficulty maintaining the representation of the new task set for switch trials over long preparation intervals) or greater advance preparation for both switch and repeat trials (e.g., children may need to maintain the representation of the relevant task set on both switch and repeat trials at long preparation intervals). In the following section, we argue that the current data are more consistent with the latter.

### Faster dissipation of task representations and increased stimulus-level interference in middle childhood

In both experiments, children's RT increased with RSI. This effect was larger for repeat trials especially at longer RSI conditions and for enhanced-cue bivalent than for univalent stimuli, resulting in a larger switch cost for univalent than bivalent stimuli. In stimulus-locked ERPs, children showed a very small switch-negativity with univalent stimuli and none at all with bivalent stimuli. Moreover, children showed less differential preparation for switch trials with bivalent stimuli, even at short preparation intervals. Together, these results suggest that children process repeat trials more like switch trials, especially under high stimulus-level interference conditions. Earlier studies also reported that children experience greater stimulus-level interference (Cepeda et al., 2001) and show a smaller repetition benefit under conditions of high stimulus interference (Cragg and Nation, 2009). Together, these findings are consistent with the notion that task representations dissipate faster in children, resulting in less repetition benefit especially at long RSI. Reduced repetition benefit would be expected to either facilitate or have little effect on preparing for switch trials. However, if task-sets dissipate more rapidly, this would have a large impact on repeat trials. If there is weaker or less consistent carry-over of activation of task-set on repeat trials, participants will need to re-load the task, much the same as on switch trials. This would predict greater similarity between switch and repeat trials especially with regard to response repetition benefit. Future work can test this directly by comparing switch and repeat trials for trials that require response repetition or response change^7^.

## Concluding remarks

Intriguingly, the pattern of results obtained here in children closely resembles those obtained in older adults. Like children, older adults typically show impairment in mixing-cost (Kramer et al., 1999; Meiran et al., 2001; Kray, 2006) but the evidence for impairment in switching costs is equivocal, with some reporting no age effect (Kray and Lindenberger, 2000; Kray et al., 2004, 2005) and others reporting age-related increase (Cepeda et al., 2001; Mayr, 2001; Kray and Eppinger, 2006). In a systematic study of task-switching in older adults using behavioral measures, decision-model parameters and ERP measures, older adults were found to have particular difficulty processing repeat trials presented in a mixed-trials block and to be more susceptible to interference from the currently-irrelevant task set (Karayanidis et al., 2011; Whitson et al., 2012). Specifically, although older adults showed greater advance preparation and higher response caution for both switch and repeat trials, they continued to show greater susceptibility to interference, especially for repeat trials during stimulus processing, response selection and response programming (Whitson et al., submitted). Thus, the absence of a switch cost difference between younger and older adults did not represent intact task-switching in older adults, but rather that older adults did not benefit from task repetition as much as younger adults did.

Taken together, the results suggest that both ends of the lifespan spectrum show greater susceptibility to post-stimulus interference especially for repeat trials, possibly due to differential decay of task representations. One possible explanation for this novel finding is that children and older adults exert cognitive control during task-switching in an inefficient or non-specific manner. Diamond (2013) argued that exerting top-down control does not always benefit performance. While top-down control is essential when learning a new task or under highly variable task conditions, when behavior is well-learnt or can be automatically triggered from external cues, top-down control can actually disrupt rather than enhance performance. In the current context, children have had considerable practice on each task and on switching between the two tasks. They show efficient preparation to switch, especially for the shorter RSI conditions, yet, show reduced repetition benefit for repeat trials. Further work is needed to test whether children and older adults unnecessarily apply controlled processes on these trials and thereby disrupt the benefit from repetition priming.

### Conflict of interest statement

The authors declare that the research was conducted in the absence of any commercial or financial relationships that could be construed as a potential conflict of interest.

## Footnotes

^1^Note that the terms general switch cost and mixing cost have not been consistently used in the literature. Here we use general switch cost to refer to differences between single task and mixed task blocks and mixing cost to refer to differences between single task blocks and repeat trials in mixed task blocks.

^2^This modification appears to have been successful: we had no participant attrition due to task difficulty. In comparison, Manzi et al. (2011) had an attrition rate of 39% in the 9–11 years group and Stoet and López (2013) had 16% in 9–16 years group with non-explicit cues.

^3^Following a trend in the literature, we use the terms univalent and bivalent stimulus conditions to refer to what Rogers and Monsell (1995) call no-crosstalk and crosstalk conditions, respectively.

^4^Across all three experiments, fewer than 5% of trials were excluded for being outside the response window.

^5^Trial numbers did not allow separate ERP waveforms to be extracted for congruent, incongruent and neutral trials. Behavioral analyses were designed to match ERP analyses.

^6^There was no main effect of Experiment or interaction between Experiment and Trial on error rate for either the children or the adults.

^7^We thank a reviewer for this suggestion. Unfortunately, there were insufficient trials in this analysis to allow a further breakdown by response hand.
